# Diethylhexyl Phthalates Is Associated with Insulin Resistance via Oxidative Stress in the Elderly: A Panel Study

**DOI:** 10.1371/journal.pone.0071392

**Published:** 2013-08-19

**Authors:** Jin Hee Kim, Hye Yin Park, Sanghyuk Bae, Youn-Hee Lim, Yun-Chul Hong

**Affiliations:** 1 Institute of Environmental Medicine, Seoul National University Medical Research Center, Seoul, Republic of Korea; 2 Environmental Health Center, Seoul National University College of Medicine, Seoul, Republic of Korea; 3 Department of Preventive Medicine, Seoul National University College of Medicine, Seoul, Republic of Korea; 4 Department of Epidemiology and Biostatistics, School of Public Health, Seoul National University, Seoul, Republic of Korea; Indiana University School of Medicine, United States of America

## Abstract

**Background:**

Insulin resistance (IR) is believed to be the underlying mechanism of metabolic syndrome and type 2 diabetes mellitus (DM). Recently, a few studies have demonstrated that phthalates could cause oxidative stress which would contribute to the development of IR. Therefore, we evaluated whether exposure to phthalates affects IR, and oxidative stress is involved in the phthalates-IR pathway.

**Methods:**

We recruited 560 elderly participants, and obtained blood and urine samples during repeated medical examinations. For the determination of phthalate exposure, we measured urinary levels of mono-(2-ethyl-5-hydroxyhexyl) phthalate (MEHHP) and mono-(2-ethyl-5-oxohexyl) phthalate (MEOHP) as metabolites of diethylhexyl phthalates (DEHP), and mono-n-butyl phthalate (MnBP) as a metabolite of di-butyl phthalate (DBP). Malondialdehyde (MDA), an oxidative stress biomarker, was also measured in urine samples. We measured serum levels of fasting glucose and insulin, and derived the homeostatic model assessment (HOMA) index to assess IR. A mixed-effect model and penalized regression spline were used to estimate the associations among phthalate metabolites, MDA, and IR.

**Results:**

The molar sum of MEHHP and MEOHP (∑DEHP) were significantly associated with HOMA (β = 0.26, *P* = 0.040), and the association was apparent among participants with a history of DM (β = 0.88, *P* = 0.037) and among females (β = 0.30, *P* = 0.022). However, the relation between MnBP and HOMA was not found. When we evaluated whether oxidative stress is involved in increases of HOMA by ∑DEHP, MDA levels were significantly associated with increases of ∑DEHP (β = 0.11, *P*<0.001) and HOMA (β = 0.49, *P* = 0.049).

**Conclusions:**

Our study results suggest that exposure to DEHP in the elderly population increases IR, which is related with oxidative stress, and that participants with a history of DM and females are more susceptible to DEHP exposure.

## Introduction

Phthalates are known to be exogenous substances which act like hormones in the endocrine system and disrupt the physiologic function of endogenous hormones to induce a range of problems in the body. Phthalates have been reported to cause antiandrogenic effects, such as decreased sperm production, infertility, as well as to harm sexual development in male infants [1]–[3]. However, the effects of phthalates on liver damage, oxidative stress, allergic symptoms, and pulmonary function were also reported recently [4]–[7]. People are easily exposed to phthalates in their daily life, as phthalates are found in literally thousands of products. Phthalates are often used in soft toys, flooring, medical equipment, paints, plastic bags, cosmetics and air fresheners [8], [9]. Because of the ubiquitous exposure, phthalates are commonly detected in human samples [5], [10]–[21].

Insulin resistance (IR) has been regarded as an important health issue because it affects the development of type 2 diabetes mellitus (DM) and metabolic syndrome, especially in elderly population [22], [23]. The number of patients with DM or metabolic syndrome has been growing very rapidly and is expected to increase worldwide in the future [22], [24], [25]. However, there is a lack of evidence whether environmental exposure to phthalates contributes to the development of IR, although there were reports on the relation between phthalate exposure and IR or DM [16], [26]–[28].

Oxidative stress has been reported to play an important role in many pathological conditions such as DM. It is defined as an impaired balance between free radical production and antioxidant capacity, resulting in excess oxidative products. Recently, a few studies have demonstrated that oxidative stress is attributable to the exposure to phthalates and contributes to development of IR [5], [29]–[32]. Even though oxidative stress is suggested as a mechanism contributing to the adverse health effects of phthalates, little information is available regarding questions of whether oxidative stress is induced from community levels of exposure to phthalates. Moreover, it is unknown whether exposure to low dose of phthalates in the environment plays a role in increasing oxidative stress in relation with the development of IR in the elderly.

Therefore, in the present study, we evaluated whether exposure to phthalates affects IR, and oxidative stress is involved in this pathway.

## Materials and Methods

### Study Population and Sampling

The Korean Elderly Environmental Panel (KEEP) study was launched in March 2008 to explore the relationships between environmental exposure and health outcomes in the elderly. A detailed description of the KEEP study design and methods are available elsewhere [33]. From its initiation until 2010, a total of 560 elderly participants, aged 60 or over visited a community elderly welfare center, as many as five times, for medical examination (twice in 2008, once in 2009, and twice in 2010). Urine samples were collected at each visit, and since fasting blood samples were obtained once per year (not obtained at the second and fifth visits), we obtained blood samples as many as three times from each individual. All urine and serum samples were placed at −20°C and −70°C, respectively, immediately following collection, and were stored until analysis for phthalate metabolites and IR indices. We also obtained detailed information of the participants using a structured questionnaire, including demographics, lifestyle habit, medical history, and a food frequency with dietary habit. The study protocol was approved by the institutional review board at Seoul National University Hospital, Seoul, Republic of Korea (IRB no. H-0804–045–241), and each study participant provided written informed consent.

### Measurement of Phthalate Metabolites

To reduce potential exposure misclassification from contamination, we measured levels of phthalate metabolites, rather than their parent compounds. Mono-(2-ethyl-5-hydroxyhexyl) phthalate (MEHHP) and mono-(2-ethyl-5-oxohexyl) phthalate (MEOHP), as metabolites of diethylhexyl phthalates (DEHP), and mono-n-butyl phthalate (MnBP) as a metabolite of di-butyl phthalate (DBP), were measured using urine samples obtained at five study visits. Phthalate metabolites were analyzed using high performance liquid chromatography tandem mass spectrometry (Agilent 6410 triple Quad LCMS, Agilent, USA) according to the previously reported procedures [5].

### Measurement of Malondialdehyde

We measured urinary levels of malondialdehyde (MDA) as an oxidative stress biomarker. Urinary MDA levels were determined by measuring thiobarbituric acid reactive substances according to the previously reported procedures [34].

### Glucose, Insulin, and HOMA

IR is characterized by elevated serum insulin concentrations in association with normal or high fasting glucose concentrations in serum. Therefore, we measured fasting glucose and insulin levels in serum collected on health examination days to evaluate IR and impaired glucose tolerance. The measurement methods for glucose and insulin levels are available in a previously reported paper [33]. We also calculated the homeostatic model assessment (HOMA) as an index of IR according to the following equation: fasting insulin (μU/ml) × [fasting glucose (mmol/l)/22.5] [35].

### Air Pollution Concentrations and Meteorological Factors

Because ambient air pollutants (PM_10_, O_3_, and NO_2_) were found to affect IR in a previous paper [33], data for PM_10_, O_3_, and NO_2_ concentration, as well as daily average outdoor temperature and dew point, were obtained from the Korea National Institute of Environmental Research and the Korea Meteorological Administration, respectively. Air pollutant concentrations and meteorological factors measured at the monitoring centers nearest to the residence of each participant were used to estimate individual exposures.

### Cotinine

Urinary cotinine levels were measured for monitoring tobacco exposure. Cotinine level was analyzed by an enzyme-linked immunosorbent assay method [34].

## Statistical Analysis

MEHHP, MEOHP, MnBP, and MDA concentrations under limit of detection (LOD) were assigned as a default value of LOD concentration divided by 2 and creatinine-adjusted levels were used to capture variation in urine concentration. Due to the high correlation between DEHP metabolites and similar characteristics (such as molecular weight) of them, we calculated the molar sum of the DEHP metabolites MEHHP and MEOHP (∑DEHP), and estimated the effects of ∑DEHP and MnBP on glucose, insulin, and HOMA indices, using a linear mixed-effect model for the panel study. In the analysis, the ∑DEHP and MnBP levels were log-transformed for normality. We evaluated three main models: model 1 adjusted for age, sex, body mass index (BMI, weight (kg)/ height^2^ (m^2^)), educational attainment, exercise and cotinine level; model 2 adjusted for model 1 variables plus air pollutants and meteorological factors which were previously reported to affect IR [33]; model 3 adjusted for model 2 variables plus dietary factors (total caloric and fat intake). Sex and exercise were treated as categorical variables and the other variables were treated as continuous variables in these models. Because IR is known to be related to DM and its prevalence may be different between males and females [16], the effect of phthalate metabolites on IR indices was evaluated separately in participants with and without a history of DM, and in males and females. To explore non-linear associations of phthalate metabolites with IR indices, a penalized regression spline of phthalate metabolites on glucose, insulin, and HOMA indices was evaluated using generalized additive mixed models (GAMM).

In the present study, the number of repeated measurements varied by participants, and this may have led to a selection bias if the loss to follow-up was not random [36]. Therefore, all analysis was conducted after weighting follow-up observations by the inverse probability of attaining a follow-up response [37] according to previously reported procedures [33].

To determine whether the concentrations of phthalate metabolites in a single urine sample can represent chronic exposure to phthalates, the relationships between a single measurement and five-sample average measurement of phthalate metabolites were estimated using Pearson correlation.

SAS version 9.2 (SAS Institute Inc., Cary, NC, USA) and R version 2.15.1 (The Comprehensive R Archive Network: http://cran.r-project.org) were used for statistical analyses.

## Results

At baseline, there was a total of 560 participants aged **≥**60, 146 (26.1%) of whom were male and 414 (73.9%) were female (Table 1). The mean number of visits was 3.3, with more visits among females (*P* = 0.038). BMI and serum insulin levels were significantly higher in females than males (*P* = 0.025 and *P* = 0.047). A history of DM, hypertension, or hyperlipidemia was reported by 91 (16.3%), 285 (50.9%), and 183 (32.7%) participants, respectively.

**Table 1:** Baseline characteristics of participants by sex.

Characteristic	Male	Female	Total
No. of participants (%)	146 (26.1)	414 (73.9)	560 (100)
No. of visit (mean ± SD)	3.1±1.3	3.4±1.4	3.3±1.4
Mean age (min-max)	71.4 (62–84)	70.5 (60–87)	70.7 (60–87)
Height [mean ± SD (cm)]	164.3±5.3	151.3±5.1	154.7±5.1
Weight [mean ± SD (Kg)]	65.7±9.8	57.1±7.4	59.3±8.1
BMI (kg/m^2^), no. (%)			
≥30	5 (3.4)	19 (4.6)	24 (4.3)
25∼<30	51 (34.9)	168 (45.2)	219 (39.1)
<25	90 (61.7)	227 (54.8)	317 (56.6)
No. of current smokers (%)	31 (21.2)	1 (0.2)	32 (5.7)
Education, no. (%)			
<High school graduate (%)	71 (49.6)	326 (81.3)	397 (73.0)
High school graduate (%)	40 (28.0)	63 (15.7)	103 (18.9)
≥College graduate (%)	32 (22.4)	12 (3.0)	44 (8.1)
Regular exercise, no. of yes (%)	90 (62.9)	253 (62.8)	343 (62.8)
Glucose (fasting levels in serum) [mean ± SD (mmol/L)] (range)	5.42±1.15 (3.50∼10.99)	5.32±1.13 (3.89∼16.32)	5.34±1.14 (3.50∼16.32)
Insulin (fasting levels in serum) [mean ± SD (μU/mL)] (range)	6.15±4.58 (0.70∼28.00)	7.14±6.41 (0.90∼76.30)	6.88±5.99 (0.70∼76.30)
HOMA (mean ± SD) (range)	1.54±1.31 (0.15∼7.39)	1.76±1.84 (0.22∼21.46)	1.70±1.72 (0.15∼21.46)
Disease history			
No. of DM (%)	25 (17.1)	66 (15.9)	91 (16.3)
No. of DM on medication (%)	24 (16.4)	64 (15.5)	88 (15.7)
No. of hypertension (%)	76 (52.1)	209 (50.5)	285 (50.9)
No. of hyperlipidemia (%)	47 (32.2)	136 (32.9)	183 (32.7)

SD, standard deviation; BMI, body mass index; HOMA, homeostatic model assessment; DM, diabetes mellitus.

Individual health examinations of all the participants during the study period totaled 1,854. We obtained 1,659 individual urinary samples among these health examinations, and measured each participant's exposures to phthalates and oxidative stress (Table 2). Levels of mean phthalate metabolite and oxidative stress were 31.99 µg/L for MEHHP, 26.15 µg/L for MEOHP, 74.41 µg/L for MnBP, and 2.12 μmol/L for MDA.

**Table 2:** Distribution of phthalate metabolite and oxidative stress biomarker levels with 5 repeated measures.

					Selected percentiles					
Biomarker	n	Mean ± SD	LOD	n< LOD (%)	10th	25th	50th	75th	90th	95th
MEHHP (μg/L)	1649	31.99±31.24	0.16	1 (0.06)	6.51	12.51	22.85	39.93	67.57	92.70
MEOHP (μg/L)	1649	26.15±26.12	0.21	1 (0.06)	4.97	9.54	18.51	33.14	56.20	77.84
MnBP (μg/L)	1649	74.41±67.16	0.27	2 (0.12)	13.32	29.40	56.57	97.18	157.68	201.72
MDA (μmol/L)	1659	2.12±1.31	0.012	4 (0.24)	0.90	1.27	1.84	2.60	3.65	4.42

SD, standard deviation; LOD, limit of detection; MEHHP, mono-(2-ethyl-5-hydroxyhexyl) phthalate; MEOHP, mono-(2-ethyl-5-oxohexyl) phthalate; MnBP, mono-n-butyl phthalate; MDA, malondialdehyde.

We estimated the effects of ∑DEHP and MnBP on glucose, insulin, and HOMA indices. Statistically significant and positive associations of ∑DEHP with HOMA were observed for all three models; Model 1 (β = 0.25; 95% confidence interval (CI): 0.02, 0.48; *P* = 0.031), model 2 (β = 0.25; 95% CI: 0.02, 0.48; *P* = 0.037), and model 3 (β = 0.26; 95% CI: 0.01, 0.51; *P* = 0.040) (Table 3). Glucose and insulin showed a similar trend with HOMA as well. However, the relationship between MnBP and IR indices was not significant for any model. Because only ∑DEHP showed a strong association with IR indices, we further analyzed only for ∑DEHP. Associations were also estimated separately among participants with and without a history of DM, and among males and females. Associations of ∑DEHP with HOMA were more apparent among participants with a history of DM (β = 0.88; 95% CI: 0.06, 1.70; *P* = 0.037), and among females (β = 0.30; 95% CI: 0.04, 0.56; *P* = 0.022) for model 3. The effect of ∑DEHP exposure on HOMA among females was consistent in females without a history of DM. Model 1 and 2 showed a similar trend with model 3 as well. However, the relationship between ∑DEHP and HOMA was not significant among participants without a history of DM, and among males for any model.

**Table 3:** Estimated associations for ∑DEHP and MnBP with IR markers.

		Glucose			Insulin			HOMA		
Model	Metabolite	β	95% CI	*P*-Value	β	95% CI	*P*-Value	β	95% CI	*P*-Value
1	∑DEHP	0.10	0.003, 0.20	0.044	0.67	−0.004, 1.34	0.051	0.25	0.02, 0.48	0.031
	MnBP	0.04	−0.06, 0.13	0.436	0.33	−0.33, 0.98	0.326	0.13	−0.09, 0.35	0.260
2	∑DEHP	0.11	0.01, 0.21	0.032	0.67	−0.02, 1.35	0.058	0.25	0.02, 0.48	0.037
	MnBP	0.05	−0.05, 0.15	0.295	0.32	−0.35, 0.99	0.351	0.13	−0.10, 0.36	0.255
3	∑DEHP	0.11	0.01, 0.22	0.033	0.70	0.01, 1.40	0.047	0.26	0.01, 0.51	0.040
	MnBP	0.06	−0.04, 0.17	0.241	0.38	−0.30, 1.07	0.273	0.16	−0.09, 0.40	0.211

β and 95% confidence interval (CI) were obtained in single pollutant models of ∑DEHP and MnBP. Changes in glucose, insulin, and HOMA indices by a log change of ∑DEHP and MnBP were obtained by model; Model 1 adjusted for age, sex, BMI, educational attainment, exercise, and cotinine level; model 2 adjusted for model 1 variables plus PM_10_ on lag day 4, O_3_ on lag day 5, NO_2_ on lag day 7, and outdoor temperature and dew point in the day; model 3 adjusted for model 2 variables plus total caloric and fat intake. ∑DEHP, molar sum of mono-(2-ethyl-5-hydroxyhexyl) phthalate (MEHHP) and mono-(2-ethyl-5-oxohexyl) phthalate (MEOHP); MnBP, mono-n-butyl phthalate; HOMA, homeostatic model assessment.

**Figure 1:**
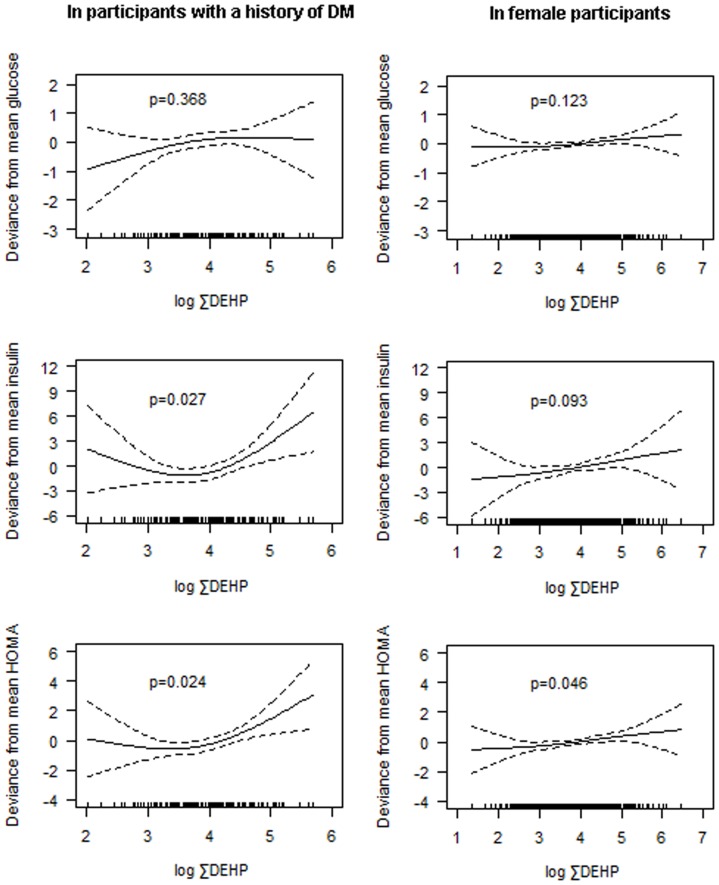
A penalized regression spline of ∑DEHP on IR markers in participants with a history of DM (n = 91) and in females (n = 414). *P*-Values were obtained using GAMM after adjusted for age, sex, BMI, educational attainment, exercise, cotinine level, PM_10_ on lag day 4, O_3_ on lag day 5, NO_2_ on lag day 7, outdoor temperature and dew point in the day, and total caloric and fat intake in participants with a history of DM, and age, BMI, educational attainment, exercise, cotinine level, PM_10_ on lag day 4, O_3_ on lag day 5, NO_2_ on lag day 7, outdoor temperature and dew point in the day, total caloric and fat intake, and a history of DM in female participants. ∑DEHP, molar sum of mono-(2-ethyl-5-hydroxyhexyl) phthalate (MEHHP) and mono-(2-ethyl-5-oxohexyl) phthalate (MEOHP); IR, insulin resistance; DM, diabetes mellitus; GAMM, generalized additive mixed model; BMI, body mass index.

To explore possible non-linearity of associations of ∑DEHP with IR indices, a penalized regression spline of phthalate metabolites on glucose, insulin, and HOMA indices was evaluated in participants with a history of DM and in females. Because associations of ∑DEHP with IR indices were different according to a history of DM, we also adjusted for a history of DM in the analysis of female participants. Significant relationships between ∑DEHP and HOMA were found in both participants with a history of DM (*P* = 0.024) and females (*P* = 0.046) (Figure 1). The relationship between ∑DEHP and IR indices was found to be non-linear in participants with a history of DM.

To determine whether the concentrations of phthalate metabolites in one spot urine samples can represent chronic exposure to phthalates, the relationships between a single measurement and five-sample average measurement of ∑DEHP and MnBP were estimated using Pearson correlation. Both of ∑DEHP and MnBP were found to have a good correlation between their single measurements and five-sample average measurements (Table 4).

**Table 4:** Correlations between single and five-sample average measurements of ∑DEHP and MnBP.

		Correlation with five-sample average	
		∑DEHP	MnBP
First-visit (n = 547)	Coefficient	0.73	0.74
	*P*-Value	<.0001	<.0001
Second-visit (n = 407)	Coefficient	0.72	0.66
	*P*-Value	<.0001	<.0001
Third-visit (n = 355)	Coefficient	0.79	0.63
	*P*-Value	<.0001	<.0001
Fourth-visit (n = 279)	Coefficient	0.68	0.56
	*P*-Value	<.0001	<.0001
Fifth-visit (n = 61)	Coefficient	0.45	0.72
	*P*-Value	0.0003	<.0001
Total (n = 1649)	Coefficient	0.72	0.67
	*P*-Value	<.0001	<.0001

∑DEHP, molar sum of mono-(2-ethyl-5-hydroxyhexyl) phthalate (MEHHP) and mono-(2-ethyl-5-oxohexyl) phthalate (MEOHP); MnBP, mono-n-butyl phthalate.

To determine whether DEHP affect IR indices by inducing oxidative stress, we analyzed the relationships among ∑DEHP, MDA, and IR indices (Table 5). After adjusting for covariates in model 3, an increase of ∑DEHP was significantly associated with MDA level (*P*<0.001) and an increase of MDA was also significantly associated with glucose and HOMA indices (*P* = 0.036 and *P* = 0.049). Particularly, the association between MDA and IR indices was found only in participants with a history of DM. However, no difference of relationship between MDA and IR indices was found in male and female participants.

**Table 5:** Estimated associations for ∑DEHP, MDA, and IR markers.

				95% CI		
Population	Exposure	Outcome	β	Lower	Upper	*P*-Value
Total participants (n = 560)						
	∑DEHP	MDA	0.11	0.06	0.15	<0.001
	MDA	Glucose	0.13	0.01	0.26	0.036
	MDA	Insulin	0.46	−0.40	1.32	0.298
	MDA	HOMA	0.31	0.001	0.62	0.049
Participants without a history of DM (n = 469)						
	∑DEHP	MDA	0.12	0.07	0.17	<0.001
	MDA	Glucose	−0.05	−0.14	0.05	0.344
	MDA	Insulin	−0.003	−0.94	0.94	0.995
	MDA	HOMA	−0.0005	−0.31	0.31	0.998
Participants with a history of DM (n = 91)						
	∑DEHP	MDA	0.04	−0.06	0.14	0.421
	MDA	Glucose	0.85	0.29	1.40	0.003
	MDA	Insulin	2.14	0.01	4.27	0.049
	MDA	HOMA	1.62	0.62	2.62	0.002

β and 95% confidence interval (CI) were obtained after adjusted for age, sex, BMI, educational attainment, exercise, cotinine level, PM_10_ on lag day 4, O_3_ on lag day 5, NO_2_ on lag day 7, outdoor temperature and dew point in the day, and total caloric and fat intake. MDA levels were log-transformed for normality. ∑DEHP, molar sum of mono-(2-ethyl-5-hydroxyhexyl) phthalate (MEHHP) and mono-(2-ethyl-5-oxohexyl) phthalate (MEOHP); MDA, malondialdehyde; IR, insulin resistance; HOMA, homeostatic model assessment; DM, diabetes mellitus.

## Discussion

This study showed that exposure to DEHP was associated with increase of IR indices in relation with oxidative stress, and participants with a history of DM and females are more susceptible to DEHP exposure.

The human body is exposed to phthalates via various routes such as medical exposures, ingestion of contaminated materials, dermal uptake from personal care products, and inhalation from outdoor and indoor air. Because biomonitoring of phthalate metabolites can avoid contamination during laboratory measurement, it has been preferably used than measurement of the parental compound [15], [38]–[40]. In our study, the urinary concentrations of MEHHP and MEOHP and MnBP, the major metabolites of DEHP and DBP, were measured as biomarkers of exposure to phthalates.

Participants in the present study were the elderly who were exposed to community levels of phthalates. Although the levels of MEHHP and MEOHP in this elderly population were similar to those in other Korean adults (29.58 and 25.06 ng/ml) [5], the levels in our participants were lower than those determined in German (47.5 and 39.7 ng/ml) and US populations (35.9 and 28.3 ng/ml) [12], [13]. However, another US population study showed lower levels of MEHHP and MEOHP than in our study; 15.1 and 7.8 ng/ml, respectively [14]. The levels of MnBP in our study were higher than those in a Korean adult study (51.84 ng/ml) and those in the National Health and Nutrition Examination Survey (mono-butyl phthalate, 40 ng/ml for males and 43 ng/ml for females) [5], [7].

Studies have linked phthalates to adverse biological effects in animals, giving rise to concerns that exposure to phthalates might cause similar effects in human beings. Although these chemicals were found at low levels in the environment, they might be able to disrupt proper functioning of the body's endocrine system to induce adverse effects. Many studies have suggested the importance of phthalates in the developmental stage or in younger individuals that are at a rapid stage of growth [2], [3], [18], [20], [21], [41], [42]. However, several studies have shown that the elderly population is also susceptible to environmental pollutants [43], [44]. For this reason, the global increase in the elderly population has given rise to concerns that environmental pollutants will have an additional burden on communities. In developed countries, people spend more than 90% of their life indoors, and phthalates are widely dispersed in the indoor environment [45]. In particular, DEHP and DBP are predominantly detected in indoor dust and air [46], [47]. Because the elderly population spends more time indoors than children and younger adults, the elderly may be more exposed to environmental toxic chemicals such as DEHP and DBP. However, it has not been demonstrated whether environmental exposure to DEHP and DBP could induce IR in the elderly, although some studies have suggested that exposure to phthalates is associated with the development of DM in adult women and in the elderly or IR in adult men [16], [26]–[28]. We added to these reports that the adverse effect of environmental exposure to DEHP on IR indices is also found in the elderly. The percent increase of HOMA index by change for interquartile-range (IQR) of ∑DEHP was found to be relatively large (≈83%, data not shown here). However, we did not find any association of MnBP with IR indices. One study of adult males conducted in the United State showed a positive association of MBP (mono-n-butyl phthalate plus mono-isobutyl phthalate) with HOMA [16]. One elderly study indicated a positive association of monoethyl phthalate and monomethyl phthalate with an increased prevalence of DM and IR [26]. However, a recently reported NHANES study showed no association of MnBP with HOMA index in a total of 3,064 women between the ages 20 and 79 years [27]. Although the study was a cross-sectional study restricted to adult women, the result supports our finding that ∑DEHP, but not MnBP, showed a positive relation with IR indices.

To figure out sensitivity regarding preexisting DM, we evaluated the effect of phthalate metabolites on IR indices in participants with and without a history of DM separately, and found a stronger association of ∑DEHP with IR indices in participants with a history of DM, indicating that participants with a history of DM may be more sensitive to ∑DEHP due to abnormal metabolism. A study indicated the possibility of a difference in the prevalence of IR in males and females due to different hormonal levels [16]. For this reason, we divided our participants into male and female groups, and then found a stronger association of ∑DEHP with IR indices in female participants, indicating that female participants may be more sensitive to ∑DEHP due to different hormonal levels. However, this finding should be interpreted cautiously because all female participants in our study were postmenopausal.

Phthalates and their metabolites have been reported to cause antiandrogenic effects [41], [42], [48]–[50]. If phthalates exert antiandrogenic effects, one might expect this to manifest over months to years, even after acute exposure to them. As the half-life of DEHP and DBP are less than 24 h [25], [38]–[40], urinary levels of these metabolites have been considered to represent acute exposures to DEHP and DBP. Moreover, due to the rapid excretion of phthalates, phthalate levels within a person can change over time. For this reason, to evaluate the representativeness of single measurements for chronic exposure, we estimated the relationships between a single measurement and five-sample average measurement of each phthalate. Both ∑DEHP and MnBP showed good correlation between single measurements and five-sample average measurements, indicating that one spot urine sample may represent chronic exposure, although these biomarkers generally represent acute exposure due to their short half-life. A study conducted by Hoppin et al. [17] showing that urinary phthalate measures are reproducible from one day to the next supports our hypothesis.

An imbalance between the oxidative stress production and antioxidant defenses may play a major role in inducing alterations in insulin signaling pathways [29]. In fact, previous studies have demonstrated close associations between reactive oxygen species (ROS) and IR [30], [31]. However, it is unclear whether current levels of environmental phthalates play a role in the development of IR by inducing oxidative stress, even though oxidative stress was reported to be increased by exposure to phthalates, and has been suggested as a potential mechanism promoting IR [5], [32]. In the present study, we estimated whether environmental exposures to phthalates affect oxidative stress in relation with the development of IR. In our study, the level of MDA was determined to be increased in dose-response relationship with ∑DEHP, and MDA was also dose-dependently associated with each IR index, particularly in participants with a history of DM. Grattagliano et al. [23] summarized that oxidative stress regulates the expression of genes governing lipid and glucose metabolism, through activation or inhibition of intracellular sensors. Therefore, our results that ∑DEHP was associated with oxidative stress, and that oxidative stress was associated with increased IR indices, support the hypothesis that exposure to phthalates increases the risk of IR by inducing oxidative stress. Many studies suggest that mitochondrial dysfunction is critical in the pathogenesis of IR [51]–[55]. As most glucose and lipid metabolism pathways are dependent on mitochondria for generating energy, when mitochondrial dysfunction is present, ATP production and oxygen consumption are impaired, and ROS production is elevated, finally leading to IR. Based on the facts that MDA is produced by oxidation of cell membranes, and oxidative phosphorylation of insulin receptor on the cell surface induces IR [55], [56], the hypothesis that mitochondrial dysfunction by exposure to phthalates induces oxidative stress leading to the production of IR is plausible. In fact, many environmental toxins have been reported to affect mitochondrial function [55]. However, this hypothesis should be confirmed through further studies in the future.

Our study had several limitations. We recruited subjects aged 60 years or older. If age modifies the effect of phthalates on IR, our results may not be generalized to younger people. We had small sample size in males compared to those in females, and in participants with a history of DM compared to those in participants without a history of DM, although repeated measure analysis increased power to our finding. Therefore, further studies are needed to confirm the difference of effect between males and females, and between participants with and without a history of DM.

In summary, we conducted repeated measurements of phthalate metabolites to evaluate the effect of environmental exposure to phthalates on IR in the elderly. Our findings suggest that exposure to phthalates increases IR, which is related with oxidative stress, particularly in participants with a history of DM.
